# Automated CT-based decoupling of the effects of airway narrowing and wall thinning on airway counts in chronic obstructive pulmonary disease

**DOI:** 10.1093/bjr/tqae211

**Published:** 2024-10-24

**Authors:** Syed Ahmed Nadeem, Xinyu Zhang, Prashant Nagpal, Eric A Hoffman, Kung-Sik Chan, Alejandro P Comellas, Punam K Saha

**Affiliations:** Department of Radiology, University of Iowa, Iowa City, IA 52242, United States; Department of Statistics and Actuarial Science, University of Iowa, Iowa City, IA 52242, United States; Department of Radiology, University of Wisconsin, Madison, WI 53792, United States; Department of Radiology, University of Iowa, Iowa City, IA 52242, United States; Department of Biomedical Engineering, University of Iowa, Iowa City, IA 52242, United States; Department of Internal Medicine, University of Iowa, Iowa City, IA 52242, United States; Department of Statistics and Actuarial Science, University of Iowa, Iowa City, IA 52242, United States; Department of Internal Medicine, University of Iowa, Iowa City, IA 52242, United States; Department of Radiology, University of Iowa, Iowa City, IA 52242, United States; Department of Electrical and Computer Engineering, University of Iowa, Iowa City, IA 52242, United States

**Keywords:** quantitative CT, artificial intelligence, causal graph analysis, airway counting, airway morphology, COPD

## Abstract

**Objective:**

We examine pathways of airway alteration due to wall thinning, narrowing, and obliteration in chronic obstructive pulmonary disease (COPD) using CT-derived airway metrics.

**Methods:**

Ex-smokers (*N* = 649; age mean ± std: 69 ± 6 years; 52% male) from the COPDGene Iowa cohort (September 2013-July 2017) were studied. Total airway count (TAC), peripheral TAC beyond 7th generation (TAC_p_), and airway wall thickness (WT) were computed from chest CT scans using previously validated automated methods. Causal relationships among demographic, smoking, spirometry, COPD severity, airway counts, WT, and scanner variables were analysed using causal inference techniques including direct acyclic graphs to assess multi-pathway alterations of airways in COPD.

**Results:**

TAC, TAC_p_, and WT were significantly lower (*P* < .0001) in mild, moderate, and severe COPD compared to the preserved lung function group. TAC (TAC_p_) losses attributed to narrowing and obliteration of small airways were 4.59%, 13.29%, and 32.58% (4.64%, 17.82%, and 45.51%) in mild, moderate, and severe COPD, while the losses attributed to wall thinning were 8.24%, 17.01%, and 22.95% (12.79%, 25.66%, and 33.95%) in respective groups.

**Conclusions:**

Different pathways of airway alteration in COPD are observed using CT-derived automated airway metrics. Wall thinning is a dominant contributor to both TAC and TAC_p_ loss in mild and moderate COPD while narrowing and obliteration of small airways is dominant in severe COPD.

**Advances in knowledge:**

This automated CT-based study shows that wall thinning dominates airway alteration in mild and moderate COPD while narrowing and obliteration of small airways leads the alteration process in severe COPD.

## Introduction

Chronic obstructive pulmonary disease (COPD) is a progressive lung disease characterised by airflow limitation. Common pathological traits of COPD include inflammatory or fibrotic narrowing of peripheral airways (bronchiolitis) and destruction of lung parenchyma (emphysema).^1^^,^^2^ CT is being increasingly used in multi-centre lung studies.^3–6^ CT-based methods enable quantification of airway morphologic features detectable at imaging resolution, which has been popularly applied to study airway-related pathophysiology in COPD and other lung diseases and to understand their impacts on disease progression and clinical outcomes.^3^ Schroeder *et al*^4^ reported that including airway measures improves the accuracy of CT-based characterization of lung function. Airway wall thinning^5^^,^^6^ as well as narrowing and obliteration of small airways^7^ in COPD have been demonstrated. Both pathways of airway alteration adversely affect CT-derived “total airway count” (TAC) in COPD.^8^

In a histologic study, McDonough *et al*^9^ found that lungs with COPD are associated with reduced airway density and cross-sectional areas of terminal bronchioles, and that small airways disappear before the onset of alveolar wall destruction. Other histologic and micro-CT-based studies have reported similar findings on airway lumen narrowing and disappearance of peripheral airways in COPD.^10^^,^^11^ Tiddens *et al*^12^ investigated airway size and wall dimensions in transversely cut cartilaginous airway sections of lung tissue obtained from COPD patients and reported reduced lumen area and a greater percentage of wall area (WA%) in COPD. While histologic methods offer accurate information at high-resolution, they are not suitable for large studies due to their invasive nature. On the other hand, CT offers an *in vivo* method to quantify anatomic and physiologic data detectable at imaging resolution and signal-to-noise ratio. Data available from CT-based large population-based studies^13–16^ have been investigated using various methods with different degrees of automation to understand disease aetiology and pathophysiology. Using a semi-automated method on total lung capacity (TLC) chest CT scans from the Genetic Epidemiology of COPD (COPDGene)^13^ study and anatomically matched airway analysis, Washko *et al*^5^ observed that both lumen narrowing and airway wall thinning increase with COPD severity, together, resulting in increasing WA% with COPD severity. Using a similar approach on both the Multi-Ethnic Study of Atherosclerosis (MESA)^16^ and Subpopulations and Intermediate Outcomes in COPD Study (SPIROMICS)^14^ chest CT data, Smith *et al*^6^ reported greater airway wall thinning with increasing COPD severity at anatomically matched airways. Using micro-CT to study specimens of centrilobular emphysema and panlobular emphysema, Tanabe *et al*^17^ observed that airway wall thickening at the level of the pre-terminal bronchioles was associated with segment shortening. Thus, actual wall loss was masked by an apparent wall thickening. Paradoxically, terminal bronchial segment shortening came in the presence of increased lung volumes. Hoffman and Weibel^18^ discussed that the apparent contradiction is explained by the concept of a tensegrity structure^19^ whereby components of a fibre continuum,^20^ maintaining the structural integrity of the lung, break. This, in turn, reduces the forces maintaining airway expansion. The objective of this study is to apply previously validated fully-automated CT-based methods of airway counting^21^^,^^22^ and wall thickness (WT) computation^23^ to decouple different pathways of airway alteration due to wall thinning, narrowing, and obliteration at different COPD severity stages.

## Methods

### Human participants and chest CT imaging

**Figure 1.**
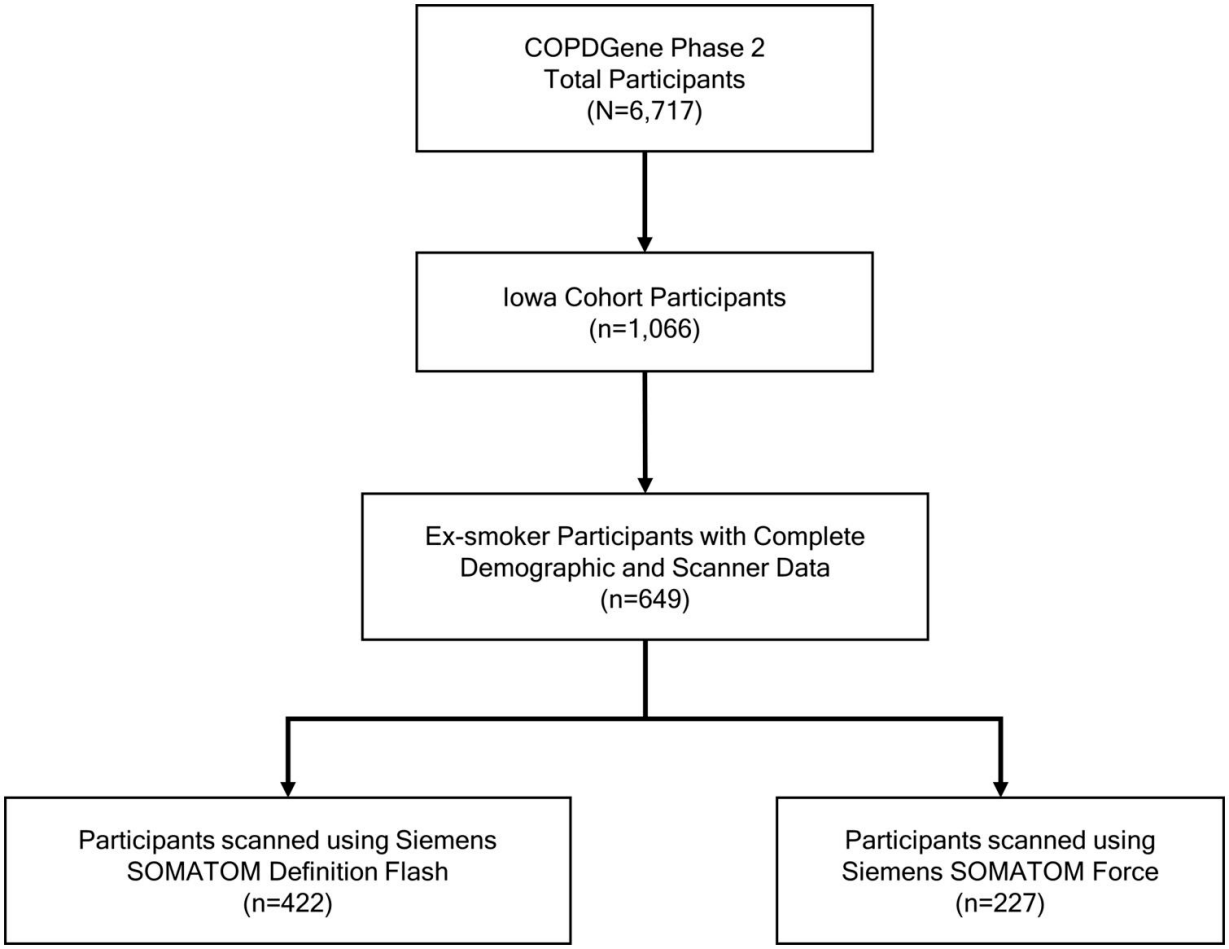
Flowchart of participant selection for this retrospective study from the Iowa cohort of the Genetic Epidemiology of Chronic Obstructive Pulmonary Disease (COPDGene) study at their first follow-up or phase 2 visits conducted between September 2013 to July 2017. Participants who were current smokers at the time of their first follow-up visits were excluded to reduce the impacts of smoking related artefacts.

Ex-smokers human participants (*n* = 649) were retrospectively selected from the Iowa cohort (*n* = 1066) of the COPDGene (ClinicalTrials.gov: NCT00608764) study^13^ (*N* = 6717) at their first follow-up visits (Figure 1). Current smokers at their first follow-up visits were excluded to reduce smoking-induced artefacts and abnormalities in airway WT. Inspiratory or TLC chest CT scans were acquired on a Siemens (Forchheim, Germany) SOMATOM Definition Flash scanner (abbreviated as “Flash”) (*n* = 422) or a Siemens SOMATOM Force scanner (abbreviated as “Force”) (*n* = 227).^13^^,^^24^ Scanner-specific imaging protocols were standardized using a quantitative CT lung assessment system (QCT-LAS).^13^^,^^24^ For both CT scanners for the Iowa cohort, inspiratory scans were acquired using 120 kVp, 200 mAs, and pitch of 1.1, and the images were reconstructed over 512 × 512 arrays with 0.75 mm slice thickness, 0.5 mm slice spacing, and approximately 0.64 mm in-plane pixel size. Previously reported repeat inspiratory chest CT scans (*n* = 37)^25^ were used to evaluate the reproducibility of our airway metrics. The Iowa cohort of the COPDGene study was approved by the University of Iowa Institutional Review Board, and written informed consent was obtained from each participant.

### Airway measurements and data analysis

Airway segmentation and branch counting in TLC chest CT scans were performed using previously validated automated methods.^21–23^^,^^26^^,^^27^ The algorithm incorporates a multi-parametric freeze-and-grow (FG) algorithm into a deep learning (DL) framework,^20^^,^^21^ where the airway tree is iteratively grown by freezing leakage regions and relaxing the threshold parameter on the DL-derived airway lumen likelihood map. Airway branches are counted by detecting bifurcations on a single-voxel skeletal representation of the airway tree after applying local scale-based pruning of false branches.^26^ A bifurcation on a skeleton is defined at a topological junction where a voxel is adjacent to more than two voxels.^28^ Topological generation or generation, in short, of a specific airway branch is defined as “1” plus the number of bifurcations encountered on the path from trachea to the specific branch. Thus, the generation of trachea is always “1”. An expert thoracic radiologist reviewed the results for forty random participants. TAC was computed as the total number of detected airways over all generations, while peripheral airway count (TAC_p_) was computed beyond the 7th generation. At a given airway branch, WT was computed by locating wall transitions on radial lines using a locally adaptive half-max method.^23^ A participant’s airway WT was estimated as the average of WT values observed at generations 7, 8, and 9. Lung size of a participant was computed as the volume of the segmented lung region available in the COPDGene database.

Age, body mass index (BMI), sex, lung size, smoking pack-years, and spirometry measures of forced expiratory volume in one second (FEV1)-to-forced vital capacity (FVC) ratio and %predicted FEV1 were incorporated into the analysis to adjust for their interdependencies with airway metrics. Global Initiative for chronic Obstructive Lung Disease (GOLD) status, defined using FEV1/FVC and %predicted FEV1, was used to characterise COPD severity groups: preserved lung function (GOLD 0); mild COPD (GOLD 1 or Preserved Ratio Impaired Spirometry [PRISm]); moderate COPD (GOLD 2); severe COPD (GOLD 3 or 4).

Two-sample *t*-tests were applied to assess group differences and statistical significance (*P* < .05). Pearson’s correlations were used for continuous variables, while biserial correlations, mathematically equivalent to Pearson correlations, were applied for dichotomous variables such as sex and CT scanner. COPD severity was represented as an ordinal variable with 0, 1, 2, and 3 for preserved lung function and mild, moderate, and severe COPD, respectively, and was treated as a continuous variable.^29^

Logarithm-transformations were applied to TAC, TAC_p_, WT, and lung size to improve the suitability of linear models, and analyses were based on these transformed values. Causal models, specifically, separate directed acyclic graphs (DAGs),^30–32^ were constructed to estimate the dependencies of TAC and TAC_p_ with different predictor and outcome variables by performing structured learning using the Grow-Shrink algorithm^33^ available in the “bnlearn” library^34^ of the software R (version: 4.2.1, https://cran.r-project.org/). During structured learning, variable relationships and their dependencies were enforced to ensure that the DAG dependencies were consistent with established biological knowledge. Specifically, no directed relationships affecting the age and sex variables were allowed. Also, the direction of the following relationships WT → TAC and COPD Severity → TAC (similarly, WT → TAC_p_ and COPD Severity → TAC_p_, for TAC_p_) were fixed to evaluate multi-pathway effects of WT and COPD Severity on CT-derived airway counts. A single-door criterion was applied to parse the direct effects of COPD, while a back-door criterion was applied for total effects. See the Online Data Supplement (Appendix S1) for details.

## Results

**Figure 2.**
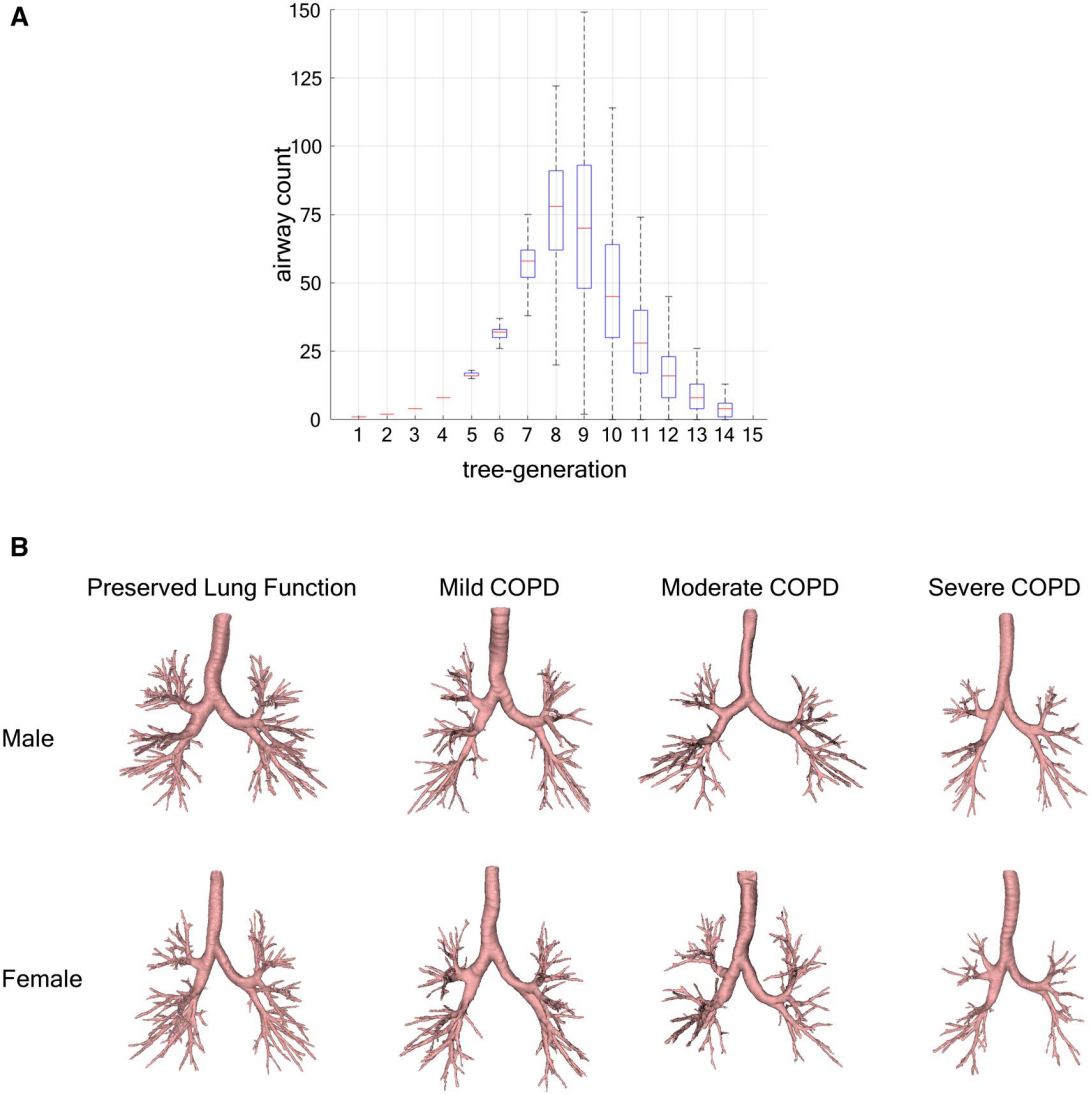
Airway tree segmentation and branch counts in inspiratory or total lung capacity chest CT scans. (A) Box-and-whisker plot of airway counts at individual tree-generations over the study population (*n* = 649, 52% male). (B) Segmented airway trees of male and female participants with preserved lung function and mild, moderate, and severe COPD with total airways counts matching to their respective group means in the study population. Preserved lung function group: participants with Global Initiative for chronic Obstructive Lung Disease (GOLD) 0; mild COPD: participants with GOLD 1 or Preserved Ratio Impaired Spirometry (PRISm), moderate COPD: participants with GOLD 2, and severe COPD: participants with GOLD 3 or 4.

The study population (*n* = 649; 52% male) included ex-smokers with preserved lung function (*n* = 362), mild COPD (*n* = 122), moderate COPD (*n* = 116), and severe COPD (*n* = 49); see Table 1 for demographic, smoking, and spirometry data. The observed values for TAC, TAC_p_, and WT spanned from 77 to 809, 13 to 680, and 0.96 mm to 1.36 mm, with mean ± std of 376.46 ± 115.96, 256.98 ± 109.37, and 1.22 ± 0.062 mm, respectively. In the expert review of 40 datasets, no false airway branch was detected; also, no segmental or sub-segmental branches were missed. Only, 0.7% (5/763) of sub-sub-segmental branches were missed. Figure 2A shows observed airway counts for the study population at different topological generations. No expert-detected spurious airway branches were reported. Anatomic generations of the airway tree are standardized as segmental, sub-segmental, and sub-sub-segmental levels along the airway paths in different lung lobes.^35^ Specifically, segmental airways are the first airway branch entering into different segments of the lung lobes, and the next two airway generations are referred to as sub- and sub-sub-segmental airways, respectively. Topological generations are defined by airway tree bifurcations, while anatomic generations are defined by entry to lung lobe segments. The observed topological generation numbers for segmental, sub-segmental, and sub-sub-segmental branches were 4.8 ± 0.7, 5.9 ± 0.8, and 6.9 ± 0.8, respectively. TAC, TAC_p_, and WT were highly reproducible in repeat CT scans^25^ with their intra-class correlation coefficient (ICC) values being 0.99, 0.99, and 0.95, respectively.

**Table 1.** Demographic characteristics, lung function, and smoking data of Genetic Epidemiology of Chronic Obstructive Pulmonary Disease (COPDGene) study participants included in this retrospective study.

Characteristics	Preserved lung function^a^	Mild COPD^a^	Moderate COPD^a^	Severe COPD^a^
No. of participants (%)	362 (55.8)	122 (18.8)	116 (17.9)	49 (7.6)
Demographic characteristics				
Age (y), mean ± std	68.7 ± 7.9	71.4 ± 7.0	72.9 ± 6.6	74.3 ± 5.8
		**(*P* < .00053)**	**(*P* < .0001)**	**(*P* < .0001)**
			(*P* = .091)	**(*P* = .0069)**
				(*P* = .18)
Female Sex, no. (%) of participants	183 (50.6)	56 (45.9)	54 (46.6)	21 (42.9)
		(*P* = .43)	(*P* = .52)	(*P* = .39)
			(*P* = 1.00)	(*P* = .85)
				(*P* = .79)
Body mass index, mean ± std	30.7 ± 5.9	31.4 ± 6.4	30.0 ± 6.6	29.3 ± 5.9
		(*P* = .29)	(*P* = .36)	(*P* = .15)
			(*P* = .11)	(*P* = .051)
				(*P* = .51)
Lung size (×10^4^ mm^2^), mean ± std	3.33 ± 0.55	3.29 ± 0.66	3.37 ± 0.56	3.54 ± 0.71
		*P* = .50	*P* = .59	*P* = .056
			*P* = .33	***P* = .036**
				*P* = .13
Smoking Pack-years, mean ± std	36.8 ± 20.8	43.5 ± 25.0	53.5 ± 25.5	58.8 ± 26.9
		**(*P* = .0091)**	**(*P* < .0001)**	**(*P* < .0001)**
			**(*P* = .0024)**	**(*P* < .0001)**
				(*P* = .25)
Spirometry (postbronchodilator) results				
FEV1/FVC, mean ± std	0.76 ± 0.10	0.68 ± 0.13	0.56 ± 0.15	0.41 ± 0.12
		**(*P* < .0001)**	**(*P* < **.**0001)**	**(*P* < **.**0001)**
			**(*P* < **.**0001)**	**(*P* < **.**0001)**
				**(*P* < **.**0001)**
FEV1 (%predicted), mean ± std	96.8 ± 16.7	79.3 ± 19.8	62.8 ± 17.2	37.9 ± 11.6
		**(*P* < **.**0001)**	**(*P* < **.**0001)**	**(*P* < **.**0001)**
			**(*P* < **.**0001)**	**(*P* < **.**0001)**
				**(*P* < **.**0001)**
CT scanner distribution				
Siemens definition flash, no. (%) of participants	247 (68.2)	71 (58.2)	73 (62.9)	31 (63.3)
		*P* = .056	*P* = .35	*P* = .59
			*P* = .54	*P* = .66
				*P* = 1.00

All participants were ex-smokers with a smoking history of at least 10 pack-years.

First, second, and third rows of *P*-values present comparisons with preserved lung function, mild COPD, and moderate COPD groups, respectively. *P*-values in bold are significant (*P* < .05).

a COPD is defined as postbronchodilator FEV1/FVC ratio of <0.7 by pulmonary function tests (spirometry). Preserved lung function consists of participants with GOLD 0, mild COPD consists of participants with GOLD 1 or PRISm, moderate COPD consists of participants with GOLD 2, and severe COPD consists of participants with GOLD 3 or 4.

Abbreviations: COPD = chronic obstructive pulmonary disease; GOLD = global initiative for chronic obstructive pulmonary disease; FEV1 = forced expiratory volume in 1 s; FVC = forced vital capacity, % predicted FEV1 = the ratio between measured FEV1 and the expected FEV1 value based on age, sex, height, and ethnicity; PRISm = preserved ratio impaired spirometry; std = standard deviation.

Participants with mild, moderate, and severe COPD were significantly older (*P* = .00053 for mild COPD and *P* < .0001 for moderate and severe COPD) and had more extensive history of smoking in pack-years (*P* = .0091 for mild COPD and *P* < .0001 for moderate and severe COPD) than those with preserved lung function (Table 1). No significant group differences were observed for sex distribution or BMI. Also, no significant differences were observed in lung size between different COPD severity groups except between mild and severe COPD groups (*P* = .036). Compared to the preserved lung function group, mild, moderate, and severe COPD groups had significantly worse (*P* < .0001) pulmonary function measurements (FEV1/FEV and %predicted FEV1), which is consistent with group definitions. Among the participants scanned on the Flash scanner and those on the Force scanner, no significant differences in age, sex, and COPD severity were observed.

### COPD and CT-based outcome variables

Summary statistics of CT-based WT, TAC, and TAC_p_ metrics are presented in Table 2. Observed WT for preserved lung function and mild, moderate, and severe COPD groups were 1.23 ± 0.05, 1.21 ± 0.06, 1.18 ± 0.06, and 1.16 ± 0.05 mm, respectively. WT significantly decreased with increasing COPD severity (*P* < .0001 for every group pair except between moderate and severe COPD groups [*P* = .030]). Participants with preserved lung function had a TAC of 417.9 ± 102.3, while those with mild, moderate, and severe COPD had TAC values of 374.1 ± 108.6, 310.9 ± 94.5, and 227.8 ± 78.8, respectively; TAC_p_ values were 294.8 ± 99.0, 255.7 ± 103.7, 195.6 ± 86.9, and 122.7 ± 68.9 for respective groups. Both TAC and TAC_p_ significantly decreased with increasing COPD severity (*P* < .0001). These results related to TAC are consistent with the representative airway tree segmentations for different COPD groups illustrated in Figure 2B.

**Table 2.** CT-based outcome variables of Genetic Epidemiology of Chronic Obstructive Pulmonary Disease (COPDGene) study participants included in this retrospective study.

Characteristics	Preserved lung function^a^	Mild COPD^a^	Moderate COPD^a^	Severe COPD^a^
No. of participants (%)	362 (55.8)	122 (18.8)	116 (17.9)	49 (7.5)
Wall thickness^b^ (mm), mean ± std	1.23 ± 0.05	1.21 ± 0.06	1.18 ± 0.06	1.16 ± 0.05
		***P* < .0001**	***P* < .0001**	***P* < .0001**
			***P* < .0001**	***P* < .0001**
				***P* = .030**
TAC,^b^ mean ± std	417.94 ± 102.35	374.13 ± 108.57	310.87 ± 94.48	227.82 ± 78.81
		***P* < .0001**	***P* < .0001**	***P* < .0001**
			***P* < .0001**	***P* < .0001**
				***P* < .0001**
TAC_p_,^b^ mean ± std	294.82 ± 98.98	255.75 ± 103.72	195.64 ± 86.90	122.71 ± 68.92
		***P* < .0001**	***P* < .0001**	***P* < .0001**
			***P* < .0001**	***P* < .0001**
				***P* < .0001**

All participants were ex-smokers with a smoking history of at least 10 pack-years.

First, second, and third rows of *P*-values present comparisons with preserved lung function, mild COPD, and moderate COPD groups, respectively. *P*-values in bold are significant (*P* < .05).

Abbreviations: COPD = chronic obstructive pulmonary disease, GOLD = Global Initiative for Chronic Obstructive Pulmonary Disease, FEV1 = forced expiratory volume in 1 second, FVC = forced vital capacity, PRISm = Preserved Ratio Impaired Spirometry.

a COPD is defined as postbronchodilator FEV1/FVC ratio of <0.7 by pulmonary function tests (spirometry). Preserved lung function consists of participants with GOLD 0, mild COPD consists of participants with GOLD 1 or PRISm, moderate COPD consists of participants with GOLD 2, and severe COPD consists of participants with GOLD 3 or 4.

b Logarithm-transformations were applied to total airway count (TAC), peripheral TAC (TACp), and airway wall thickness for subsequent analysis to improve the linear relationship between them and airway tree generation.

### Relationships between predictors and CT-based outcome variables

**Figure 3.**
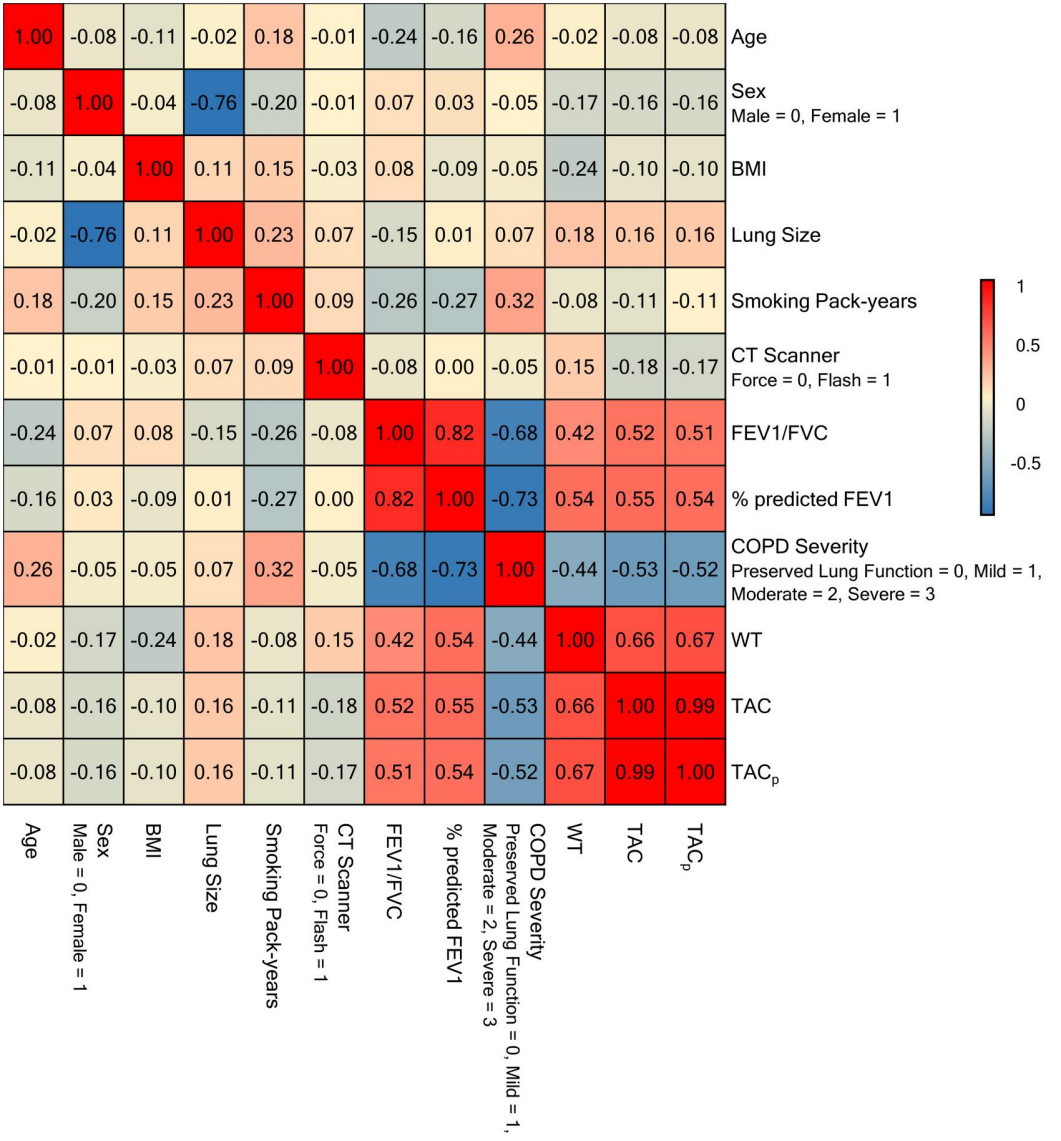
Pearson correlations between different demographic, smoking, lung function, and CT-derived variables. Note that biserial correlations, mathematically equivalent to Pearson correlations, were applied for dichotomous variables such as sex and CT scanner. Chronic obstructive pulmonary disease (COPD) severity was represented as an ordinal variable, specifically, 0 for preserved lung function, 1 for mild COPD, 2 for moderate COPD, and 3 for severe COPD, and was treated as a continuous variable. BMI: body mass index. Lung size was computed as the maximum axial lung cross-sectional area. Airway wall thickness (WT) was computed as the average of the mean airway wall thickness at generations 7-9. Preserved lung function group: participants with global initiative for chronic obstructive lung disease (GOLD) 0; mild COPD: participants with GOLD 1 or preserved ratio impaired spirometry (PRISm), moderate COPD: participants with GOLD 2, and severe COPD: participants with GOLD 3 or 4.

Figure 3 presents correlations among different variables. Negligible correlations of the CT scanner variable with demography, smoking history, lung function, and COPD severity suggest no population bias between the scanners. Females had lower lung size (*r* = −0.76), TAC (*r* = −0.16), TAC_p_ (*r* = −0.16), and WT (*r* = −0.17) and less severe COPD (*r* = −0.05) as compared to males. A positive correlation of age with COPD severity (*r* = 0.26) is consistent with Table 1. High negative correlations between COPD severity and different spirometry measures were expected. TAC and TAC_p_ were very highly correlated (*r* = 0.99), while WT showed moderate positive correlations (*r* = 0.66 and 0.67 for TAC and TAC_p_, respectively). Both TAC and TAC_p_ showed moderate positive correlations with spirometric lung function measures and moderate negative correlations with COPD severity. Specifically, the r values for TAC with FEV1/FVC, %predicted FEV1, and COPD severity were 0.52, 0.55, and −0.53, respectively, and those values for TAC_p_ were 0.51, 0.54, and −0.52, respectively. Relatively lower correlations were observed for airway WT with spirometry measures of FEV1/FVC (*r* = 0.42), %predicted FEV1 (*r* = 0.54), and COPD severity (*r* = −0.44).

### Direct and indirect effects of COPD on airway counts and wall thickness

**Figure 4.**
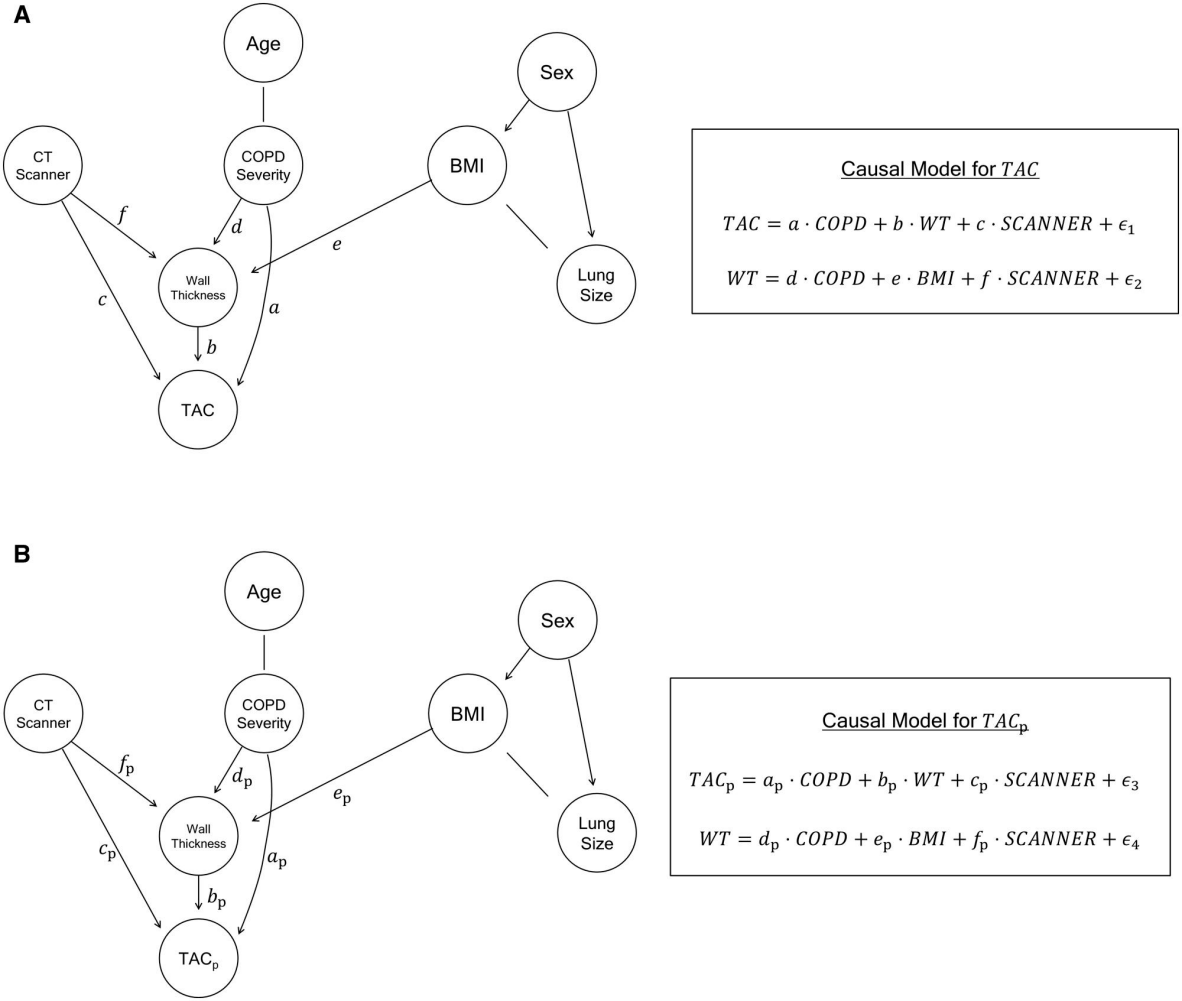
Causal inference graphs for CT-derived (A) total airway count (TAC) and (B) peripheral TAC (TAC_p_) beyond the 7th airway tree generation. BMI: body mass index; COPD: chronic obstructive pulmonary disease. Lung size was computed as the maximum axial lung cross-sectional area. Airway wall thickness (WT) was computed as the average of the average airway wall thickness at generations 7-9.

Both TAC and TAC_p_ produced the same DAG structure suggesting two simultaneous COPD-associated pathways affecting total and peripheral airway counts (Figure 4). Specifically, two paths from the “COPD Severity” node to “TAC” were identified. The indirect effect (bd in Figure 4) is reflected by the path *via* “WT”. Thus, the indirect effect represents the COPD-imaging interaction that links to wall thinning and technical detectability of airways in CT scans. The direct effect (a in Figure 4) is assessed along the path from “COPD Severity” to “TAC”. It characterises a second pathology-imaging interaction, which may be attributed to missing airways due to narrowing and obliteration of small airways related to parenchymal destruction and disruption of the lung’s structural tensegrity, leading to loss of lung elastic recoil. Compared to the preserved lung function group, mild, moderate, and severe COPD groups had direct effect coefficients of −0.047, −0.14, and −0.39 and indirect effect coefficients of −0.086, −0.19, and −0.26, respectively, for TAC. Findings on TAC_p_ were similar. The direct and indirect effect coefficients for TAC_p_ for these three COPD groups were −0.047, −0.20, −0.61, and −0.14, −0.30, −0.41, respectively. Following the log transformation of TAC and TAC_p_ during analysis, a coefficient value α  was translated into a percentage change in TAC or TAC_p_ compared to participants with preserved lung function using $\left(e^{\alpha }-1\right)\times 100\%$. Furthermore, the DAGs elucidate that BMI, WT, and CT scanner are confounding variables either directly or indirectly, *via* WT, affecting TAC (or, TAC_p_).

**Figure 5.**
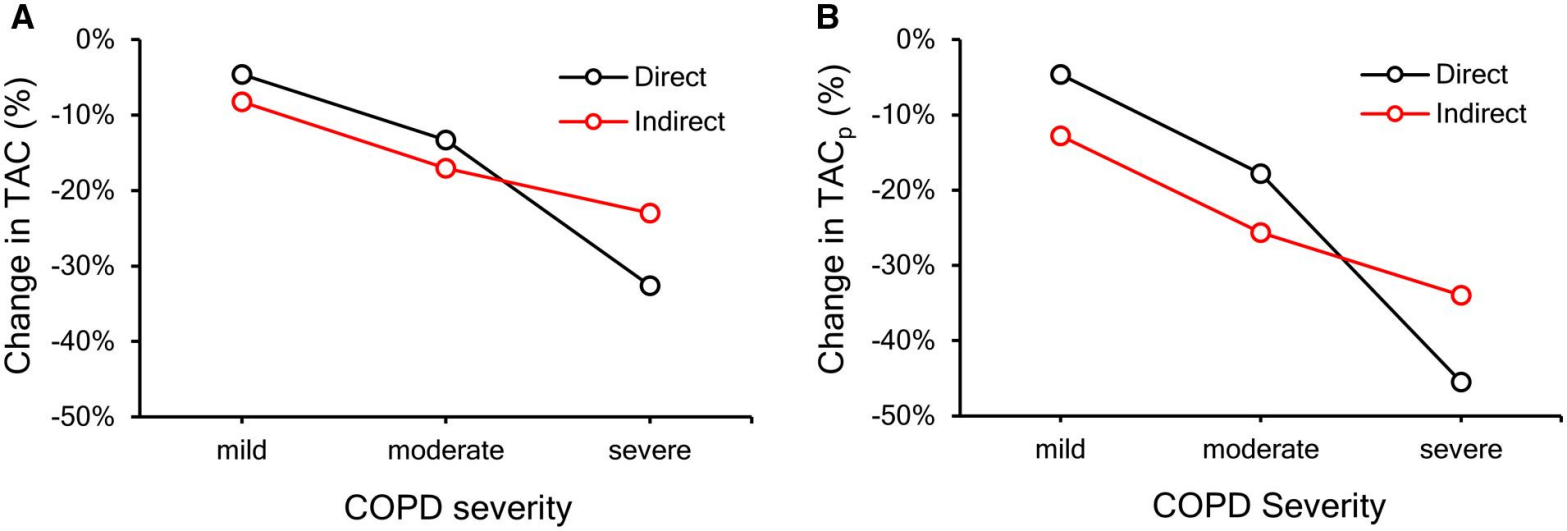
The effect of chronic obstructive pulmonary disease (COPD) on CT-derived (A) total airway count (TAC) and (B) peripheral TAC (TAC_p_) beyond the 7th generation in terms of percentage change. Direct effects of COPD on CT-derived airway counts may be attributed to narrowing and obliteration of small airways due to emphysema-related loss of lung elastic recoil, while indirect effects are attributed to missing airways due to wall thinning. Mild COPD: participants with global initiative for chronic obstructive lung disease (GOLD) 1 or preserved ratio impaired spirometry (PRISm), moderate COPD: participants with GOLD 2, and severe COPD: participants with GOLD 3 or 4.

Figure 5 presents estimated changes in TAC and TAC_p_ due to the direct and indirect effects of COPD at different disease severity. The direct effect of COPD contributes to 4.59% and 13.29% declines in TAC for mild and moderate COPD groups, respectively, which were less in magnitude than 8.24% and 17.01% declines for respective groups due to the indirect effect. In severe COPD, the direct effect of COPD of 32.58% decline in TAC was stronger than the indirect effect of 22.95%. Findings for TAC_p_ were similar except that the magnitudes of decline in both direct and indirect effects as well as the differences between direct and indirect effects were enhanced for all three COPD severity groups. Additionally, WT was found to be impacted by COPD severity, BMI, and CT scanner and was indirectly impacted by age through COPD severity and sex through BMI. It was observed that the CT scanner variable directly affects TAC, TAC_p_, and WT. Sex was found to affect BMI and lung size, while BMI and lung size, as well as age and COPD severity were related without any causal direction.

## Discussion

The fully-automated CT-based method produced no false or spurious airway branches and delivered high accuracy of branch detection. These observations are consistent with our previous findings on different datasets that the automated method is accurate, repeatable, and generalizable.^21^^,^^22^ The performance of the method creates an opportunity to retrospectively apply it to multi-centre CT-based pulmonary studies investigating airway-related pathophysiology in lung diseases. If needed, the DL component of the algorithm may be efficiently retrained using transfer learning,^36^^,^^37^ where the ground truth may be generated using the intensity-based FG method^21^ that is computationally slow but requires no training.

Two directed paths from COPD severity node to TAC (or, TAC_p_) representing image-derived airway counts are found on the DAGs of Figure 4 derived by causal inference analysis of automated CT-based measures of airway metrics. These paths represent two discrete pathologic processes altering CT-derived airway counts in COPD are found: (i) narrowing and obliteration of small airways and (ii) failure to detect airways in CT due to wall thinning. Peripheral airway destruction^1^^,^^11^ and luminal narrowing in the presence of late-stage COPD-associated lung volume expansion^18^ have been established in the literature explaining the first pathway. This luminal narrowing has been attributed to loss of lung elastic recoil associated with parenchymal destruction and disruption of the lung’s tensegrity structure.^17–20^^,^^38^ The second pathway is attributed to airway wall thinning in COPD,^5^^,^^6^ leading to failure to detect small airways in CT. Both of these pathologies interplay with the limits of CT spatial resolution and airway wall and lumen detectability serving to truncate the segmented airway tree and limiting the TAC. The current study establishes a method to decouple and weigh the impacts of the two pathological pathways of airway alterations in COPD using CT-derived metrics. This method may be helpful in tracking biological processes at different stages of COPD or other pulmonary diseases *via* automated quantitative CT. Specifically, our findings suggest that, in mild and moderate COPD, the negative impact on airway count through wall thinning may be the dominant phenomenon relative to the direct effect of COPD attributed to narrowing and obliteration of small airways due to emphysema-associated disruption of the lung fibre network. Relative magnitudes of negative impacts of the two pathways flip in the later stages of COPD. These findings are novel and particularly important because the pathway attributed to the narrowing and obliteration of small airways is associated with significantly higher rates of exacerbation, hospitalization, and 5-year mortality as compared to mild and moderate COPD.^2^ Following the DAGs, BMI, WT, and CT scanner are confounding variables affecting direct and indirect causal effects of COPD on airway counts. Therefore, the adjustment of these variables, as defined in the equations in Figure 4, is essential for obtaining unbiased estimates of direct and indirect causal effects of COPD on image-derived airway counts.

The observations presented here decouple two underlying airway alteration pathways in COPD and quantitatively weigh their impacts at different stages of COPD using a CT-based automated method and a causal inference model. However, it is worth mentioning that the CT-based airway count and morphologic metrics may not express the true anatomy of the airway tree; instead, they represent image-derived characterization of a pathologic process.

CT scanner technology and resolution have been improving, and one of the aspects of our causal inference analysis was to evaluate the effects of CT scanners on detection of the airways and measurement of WT. The observation that there was no age, sex, and COPD severity bias between participants imaged in two scanners eliminates population-related confounding effects of scanners on airways measures in the causal inference analysis.

CT-derived WT of peripheral airways significantly decreased with increasing COPD severity, which is consistent with the CT-derived findings of Washko *et al*^5^ and Smith *et al*,^6^ where greater loss in airway WA was observed with increasing COPD severity at anatomically matched segmental and sub-segmental airways. Following the negative association of WT with COPD severity, observed strong negative correlations of WT with spirometry measures of lung function were expected. The relationships between COPD severity and TAC are consistent with previous CT-derived findings.^8^^,^^9^^,^^11^ CT-derived TAC_p_ significantly decreased with increasing COPD severity. Peripheral airway counts of mild, moderate, and severe COPD groups were significantly lower than that of the preserved lung function group, which is consistent with histologic observations on loss of small and peripheral airways at the onset of COPD.^7^

Besides COPD severity and lung function, higher airway counts and WT were observed among males, which is consistent with the known dependence of COPD susceptibility on sex observed in spirometry-based studies.^2^ However, reduced CT-derived airway counts in females may be primarily attributed to reduced lung size and concomitant generation-matched airway wall and lumen dimensions in females, which is consistent with CT-based observations of others.^3^ Observed weak negative correlations of airway counts with ageing may be explained by increased airway destruction with ageing as demonstrated histologically^39^ and loss of lung elastic recoil.^40^^,^^41^

Smoking pack-years, a well-known risk factor of COPD,^2^ were expectedly negatively correlated with WT and airway counts. WT and airway counts were weakly and negatively correlated with BMI, and BMI had a direct negative effect on WT in the causal inference analysis. Conflicting findings on the relationship between BMI and WT have been reported in the literature using CT-based studies,^42^^,^^43^ which may be partially attributed to imaging challenges and bias in CT-derived metrics for participants with high BMI. Increase in generation-matched airway size in larger lungs^3^ improves airway detectability causing positive correlations between lung size and airway metrics as observed here; weakness in correlations may be explained by the presence of different confounding factors including sex, COPD severity, age, and smoking history.

Our results on associations of airway counts and morphology with COPD severity, sex, age, BMI, and smoking history are consistent with histologic and semi-automated CT-based findings and extend those to peripheral airways using a CT-based automated method.

A limitation of this study is that the participants were all former smokers and mostly non-Hispanic Caucasians, and therefore, results may not be extended to a broader population. Also, the COPDGene Iowa cohort represents a rural population, and results may vary in an urban population with different lifestyles and exposures.

This automated CT-based study decouples different pathways of airway alteration due to wall thinning, narrowing, and obliteration at different COPD severity stages. Specifically, the study shows that wall thinning is a dominant contributor to TAC loss in mild and moderate COPD, while narrowing and obliteration of small airways is enhanced and leads in severe COPD. Also, it demonstrates the links between total and peripheral airway counts and morphology with COPD severity, sex, ageing, and smoking history.

## Supplementary Material

tqae211_Supplementary_Data

## Data Availability

Deidentified data from the COPDGene study is publicly available on request. Further details of the COPDGene study are available online.
