# Supplementary material for: Automated CT-based decoupling of the effects of airway narrowing and wall thinning on airway counts in chronic obstructive pulmonary disease
Source: Br J Radiol. 2024 Oct 24;98(1165):150–9. doi: 10.1093/bjr/tqae211 (PMC11652725; doi:10.1093/bjr/tqae211)
Supplement: tqae211_Supplementary_Data [file tqae211_supplementary_data.docx]

**Title:** Automated CT-based Decoupling of the Effects of Airway Narrowing and Wall Thinning on Airway Counts in Chronic Obstructive Pulmonary Disease

**Running title:** Automated CT-based Decoupling of Airway Alterations in COPD

**Online Data Supplement**

Appendix I

**Causal Inference Analysis**

1. Introduction of Causal Graph

Causal graph is a powerful tool for causal inference of depicting causal relationships among multiple variables. Directed acyclic graph (DAG) is the most commonly used causal graph. A directed graph $\left( G, E \right)$comprises of a finite set of nodes $G$, each of which represents a distinct random variable, and a collection $E\subset G\times G$ of ordered pairs of distinct nodes. An ordered pair $\left( a,b \right)\in E$ denotes a directed edge in a DAG extending from node $a$ to node $b$, in which case, $a$ is said to be a parent of $b$. Such directed edges represent the causal relationships between these variables. Being a directed acyclic graph, a DAG contains no directed cycles, i.e., there exists no finite sequence of directed edges $\left\langle\left. \left( a_{i},a_{i+1} \right) \right|i=1,\ldots, m-1 \right\rangle$such that $a_{1}=a_{m}$. A DAG $\left( G, E \right)$ depicts the conditional dependency structure among the variables in $G$ if the joint distribution of $G$is Markov w.r.t. the DAG, i.e., their joint probability density function (pdf) or joint probability mass function (pmf) can be factorized as a product of the conditional pdf (or, pmf) of each node given its parents. The acyclic property ensures that there are no direct or indirect causal loops, which is crucial for inferring causal relationships. A DAG can be constructed solely based on subject-matter considerations or estimated from data. The latter approach can also incorporate some subject-matter knowledge, which promotes meaningful causal diagrams. From a given DAG, the presence or absence of direct causal relationships between pairs of variables are immediately known, and their implicit indirect causal relationships can be readily deduced. More importantly, it shows which variables may confound a causal relationship of interest, and therefore, by adjusting these confounding variables via linear regression, both the linear direct causal and indirect causal effects of interest can be quantitatively inferred from data. In order to implement causal inference using causal graphs, there are mainly two steps: DAG construction and causal effects identification.^E1^ These steps are briefly described in the following.

1. Structure Learning

To uncover the causal relationships among a set of variables, the fundamental task is structure learning of DAG, i.e., inferring the most likely causal structure that best fits the observed data.

Instead of manually setting the graph solely based on expert domain knowledge, DAG structure learning may be done via a data-driven approach. There are mainly two kinds of DAG structure learning algorithms: constraint-based methods and score-based methods. In this paper, we use the Grow-Shrink algorithm,^E2^ which is a constraint-based DAG learning method. To ensure that the learned DAG was consistent with established biological knowledge, we also incorporate expert domain knowledge in structure learning through arc (directed edge) priors and blacklists. Specifically, the following directed relationships were blacklisted: CT scanner→age, sex→age, and COPD severity→age and the direction of the following arcs were fixed: WT→TAC and WT→TAC_p_.

Our data involves both continuous and discrete variables. However, in a hybrid Bayesian network, a continuous node is not allowed to be the parent of a discrete node, and such restriction may result in some biologically infeasible relationships. To address this constraint, the ordinal discrete variable, COPD severity, was treated as a continuous variable in structure learning. A recent paper^E3^ proposed this strategy, and both theoretically and empirically proved its reliability. Our learned DAGs for TAC and TAC_p_ are shown in Figure 4.

1. Causal Effect Identification

After the DAG structure has been learned, we can examine the paths between variables to determine which variables are causally related, understand the direction and strength of causal effects, and distinguish between direct and indirect causal effects. We shall assume linear causal effects. Thus, linear regression facilitates the main tool to adjust for confounding variables and quantify causal relationships. A direct effect refers to an effect of a causal variable on an outcome variable that is not mediated by other variables in the model. On the other hand, an indirect effect refers to an effect of a causal variable on an outcome variable that operates through one or more intermediate variables in a causal pathway. In a DAG, a direct effect is represented by a direct arrow connecting the two variables, while an indirect effect is represented by a chain of arrows connecting the cause, intermediate, and outcome variables.

For the current study, we consider the direct effect of COPD to TAC (or, TAC_p_) and the indirect effect of COPD to TAC (or, TAC_p_) via WT. Based on the DAGs (Figure 4), direct and indirect causal effects were estimated by regression under predefined criteria. Specifically, we applied the single-door criterion for the direct effect and the back-door criterion for the total effect and obtained the following relationships for TAC (similarly, TAC_p_) as shown in Figure 4:

$$\begin{matrix} TAC & =a\cdot COPD+b\cdot WT+c\cdot SCANNER+\epsilon_{1} \\ WT & =d\cdot COPD+e\cdot BMI+f\cdot SCANNER+\epsilon_{2} \end{matrix}$$

Thus, the effect of COPD to TAC (or, TAC_p_) can be decomposed into direct effect, which is estimated by $a$, and indirect effect, which is estimated by $bd$. Following the log transformation of TAC and TAC_p_ during our analysis, a coefficient value $\alpha$ was translated into the percentage change in TAC or TAC_p_ compared to participants with preserved-lung-function using $\left( e^{\alpha}-1 \right)\times100\%$, i.e., $\left( e^{a}-1 \right)\times100\%$ for direct effect and $\left( e^{bd}-1 \right)\times100\%$ for indirect effect.

# References

1. Pearl J. Models, Reasoning and Inference. Cambridge, UK: Cambridge University Press; 2000.
2. Margaritis D. Learning Bayesian network model structure from data. School of Computer Science, Carnegie Mellon University Pittsburgh, PA, USA; 2003.
3. Zhu W, Nguyen NLC, Cripps S. Structure learning for hybrid Bayesian networks. arXiv preprint arXiv:220601356 2022.
